# The CB_1_ cannabinoid receptor signals striatal neuroprotection via a PI3K/Akt/mTORC1/BDNF pathway

**DOI:** 10.1038/cdd.2015.11

**Published:** 2015-02-20

**Authors:** C Blázquez, A Chiarlone, L Bellocchio, E Resel, P Pruunsild, D García-Rincón, M Sendtner, T Timmusk, B Lutz, I Galve-Roperh, M Guzmán

**Affiliations:** 1 Centro de Investigación Biomédica en Red sobre Enfermedades Neurodegenerativas (CIBERNED) and Instituto Ramón y Cajal de Investigación Sanitaria (IRYCIS), Madrid, Spain; 2 Department of Biochemistry and Molecular Biology I, School of Biology, Complutense University, and the Instituto Universitario de Investigación Neuroquímica (IUIN), Madrid, Spain; 3 Institute of Gene Technology, Tallinn University of Technology, Tallinn, Estonia; 4 Institute of Clinical Neurobiology, University of Würzburg, Würzburg, Germany; 5 Institute of Physiological Chemistry, University Medical Center of the Johannes Gutenberg University Mainz, Mainz, Germany

## Abstract

The CB_1_ cannabinoid receptor, the main molecular target of endocannabinoids and cannabis active components, is the most abundant G protein-coupled receptor in the mammalian brain. In particular, the CB_1_ receptor is highly expressed in the basal ganglia, mostly on terminals of medium-sized spiny neurons, where it plays a key neuromodulatory function. The CB_1_ receptor also confers neuroprotection in various experimental models of striatal damage. However, the assessment of the physiological relevance and therapeutic potential of the CB_1_ receptor in basal ganglia-related diseases is hampered, at least in part, by the lack of knowledge of the precise mechanism of CB_1_ receptor neuroprotective activity. Here, by using an array of pharmacological, genetic and pharmacogenetic (*designer receptor exclusively activated by designer drug*) approaches, we show that (1) CB_1_ receptor engagement protects striatal cells from excitotoxic death via the phosphatidylinositol 3-kinase/Akt/mammalian target of rapamycin complex 1 pathway, which, in turn, (2) induces brain-derived neurotrophic factor (BDNF) expression through the selective activation of *BDNF* gene promoter IV, an effect that is mediated by multiple transcription factors. To assess the possible functional impact of the CB_1_/BDNF axis in a neurodegenerative-disease context *in vivo*, we conducted experiments in the R6/2 mouse, a well-established model of Huntington's disease, in which the CB_1_ receptor and BDNF are known to be severely downregulated in the dorsolateral striatum. Adeno-associated viral vector-enforced re-expression of the CB_1_ receptor in the dorsolateral striatum of R6/2 mice allowed the re-expression of BDNF and the concerted rescue of the neuropathological deficits in these animals. Collectively, these findings unravel a molecular link between CB_1_ receptor activation and BDNF expression, and support the relevance of the CB_1_/BDNF axis in promoting striatal neuron survival.

The CB_1_ receptor is the most abundant G protein-coupled receptor in the mammalian brain.^1^ This receptor is engaged by endocannabinoids, a family of prostanoid-like neural messengers, as well as by Δ^9^-tetrahydrocannabinol (THC), the main active component of the hemp plant *Cannabis sativa*.^1, 2, 3^ Endocannabinoid signaling serves as a major feedback mechanism aimed at preventing excessive pre-synaptic activity, thereby tuning the functionality and plasticity of many synapses. In particular, the CB_1_ receptor is very highly expressed in GABAergic terminals of the forebrain, where it mediates endocannabinoid-dependent inhibition of GABA release.^1^

In concert with this well-established neuromodulatory function, one of the most remarkable biological actions of the CB_1_ receptor is to prevent neuronal death. This effect has been reported in many different animal models of acute brain damage and chronic neurodegeneration, and has raised hope about the possible clinical use of cannabinoids as neuroprotective drugs.^1, 4, 5, 6^ However, the assessment of the physiological relevance and therapeutic potential of the CB_1_ receptor in neurological diseases is hampered, at least in part, by the lack of knowledge on the precise molecular mechanisms of CB_1_ receptor neuroprotective activity.^5, 7^ It is well established that CB_1_ receptor engagement inhibits excitotoxic neurotransmission by blunting pre-synaptic glutamate release, and this has been put forward as a major event underlying CB_1_ receptor-mediated neuroprotection.^1, 6, 8, 9^ However, it is plausible that additional processes contribute to the neuroprotective activity of the CB_1_ receptor. Specifically, studies conducted in the mouse and rat brain have reported a close association between CB_1_ receptor activity and the expression of brain-derived neurotrophic factor (BDNF),^5, 7^ one of the master neurotrophins in the mammalian forebrain.^10^ Moreover, acute intravenous administration of THC to healthy volunteers increases BDNF levels in the serum,^11^ thus suggesting that a CB_1_/BDNF connection could also exist in humans.

A putative CB_1_/BDNF connection might be particularly relevant in the striatum, and influence their related motor disorders (e.g., Huntington's disease (HD) and Parkinson's disease), as, for example, (1) the CB_1_ receptor is highly expressed in medium-sized spiny neurons (MSNs), the cells that constitute ~90% of total striatal neurons, and plays a key role in the control of motor behavior by basal ganglia circuitry;^4, 12^ (2) BDNF and its high-affinity receptor, TrkB, exert a pivotal function in MSN generation, survival and plasticity;^13, 14, 15^ and (3) striatal CB_1_ receptor,^16^ BDNF^17^ and TrkB^18^ expression declines along disease progression in animal models of HD, and restoration of CB_1_ receptor,^19^ BDNF^20^ or TrkB^21, 22^ function prevents HD-like neurodegeneration.

In spite of these concerted changes in CB_1_ receptor activity and BDNF expression, no causative link between the two events has been defined yet. Hence, here we sought to establish a molecular connection between CB_1_ receptor activation and BDNF expression in the striatum, and to assess the possible neuroprotective relevance of this putative CB_1_/BDNF axis.

## Results

### The CB_1_ receptor protects cultured striatal cells from excitoxicity via PI3K/Akt/mTORC1/BDNF

**Figure 1:**
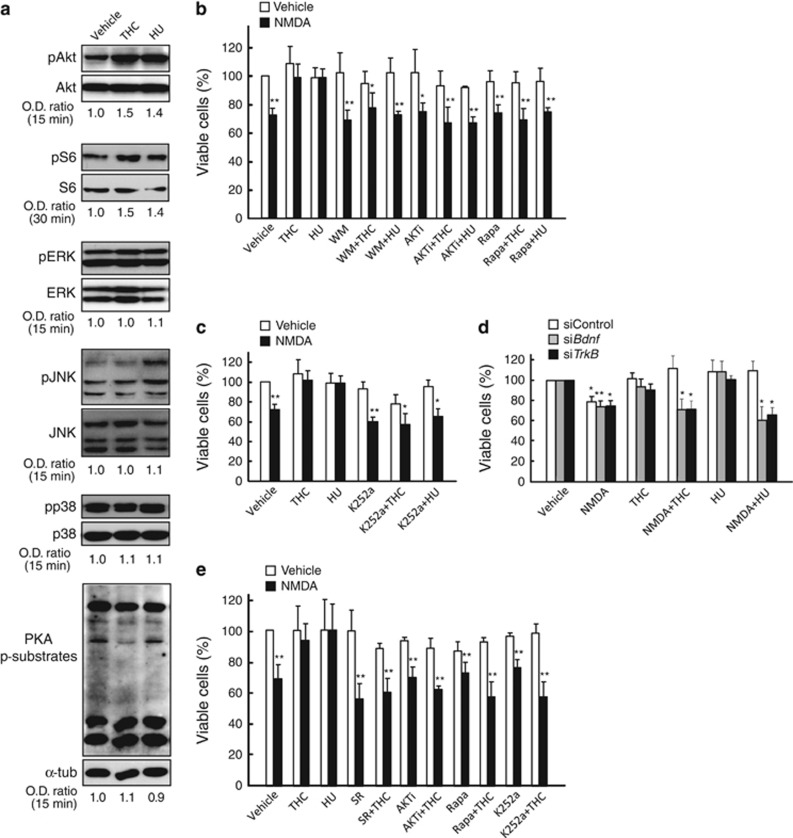
The CB_1_ receptor protects cultured striatal cells from NMDA-induced excitoxicity via PI3K/Akt/mTORC1/BDNF. (**a**) STHdh^Q7/Q7^ cells were incubated for the times indicated with vehicle, 0.5-*μ*M THC or 10-nM HU-210. Cells were lysed and western blot analyses were conducted. Quantification of mean optical density (O.D.) values relative to those of loading controls (respective total proteins, or *α*-tubulin in the case of PKA phosphorylated substrates) as well as representative blots are shown (*n*=3–4 experiments). (**b**, **c**) STHdh^Q7/Q7^ cells were preincubated for 5 h in Locke's solution with or without 1-mM NMDA together with vehicle, 0.5-*μ*M THC, 10-nM HU-210, 0.2-*μ*M wortmanin, 0.1-*μ*M Akti-1/2, 30-nM rapamycin and/or 25-nM K252a, and subsequently incubated for 24 h in NMDA-free medium. Relative cell viability is shown (*n*=6–8 experiments). (**d**) STHdh^Q7/Q7^ cells were transfected with a non-targeted siRNA or with siRNAs directed against *BDNF* or *TrkB*, and subsequently incubated for 5 h with or without NMDA, THC and/or HU-210 as in **b**. Relative cell viability is shown (*n*=4–6 experiments). (**e**) Primary mouse striatal neurons were incubated for 30 min in Locke's medium with or without 50-*μ*M NMDA, together with vehicle, 0.3-*μ*M THC, 10-nM HU-210, 0.25-*μ*M SR141716, 0.1-*μ*M Akti-1/2, 20-nM rapamycin and/or 10-nM K252a, and subsequently incubated for 2 h in NMDA-free medium. Relative cell viability is shown (*n*=4–6 experiments). Data were analyzed using ANOVA with *post hoc* Student-Newman-Keuls test. *P<0.05 and **P<0.01 from the corresponding vehicle-treated cells

The CB_1_ cannabinoid receptor is a pleiotropic G protein-coupled receptor that modulates various pathways potentially involved in the control of cell survival such as phosphatidylinositol 3-kinase (PI3K)/Akt, mitogen-activated protein kinases (extracellular signal-regulated kinase (ERK), c-Jun *N*-terminal kinase (JNK) and p38) and cAMP/protein kinase A (PKA).^23^ To study the mechanism of CB_1_ receptor-mediated neuroprotection, we first used STHdh^Q7/Q7^ mouse striatal neuroblasts, a widely used neuron-like cell line^24^ that expresses functional CB_1_ receptors.^19^ Cells were incubated with two paradigmatic cannabinoid receptor agonists (THC, the major active ingredient of marijuana, and HU-210, a highly-potent synthetic derivative of THC) and evaluated how the aforementioned pathways were affected. Exposure of cells to cannabinoids led to a rapid (15 min) and transient (15–30 min) phosphorylation (activation) of Akt, which was followed by a transient (30 min) phosphorylation (activation) of ribosomal S6 protein, a canonical substrate of the Akt/mammalian target of rapamycin complex 1 (mTORC1) pathway (Figure 1a and Supplementary Figure S1a). This effect was dose dependent (Supplementary Figure S1b). In contrast, the phosphorylation status of ERK, JNK, p38 and PKA substrates was not changed by cannabinoids (Figure 1a).

These findings prompted us to test the involvement of the PI3K/Akt/mTORC1 pathway in CB_1_ receptor-mediated neuroprotection. We used STHdh^Q7/Q7^ cells exposed to the well-established excitotoxin *N*-methyl-d-aspartate (NMDA) because the CB_1_ receptor is known to exert cytoprotection in that experimental system.^19^ In agreement with some authors,^25, 26^ but, owing to unobvious reasons, in disagreement with others,^27^ we could readily detect transcripts encoding NMDA receptor subunits in STHdh^Q7/Q7^ cells (threshold cycle (Ct) values: NR1, 35; NR2A, 35; NR2B, 27; NR2C, 32; and NR2D, 34). These values support that NR2B and NR2C might be responsible for NMDA-induced responses in STHdh^Q7/Q7^ cells, and that these cells express very low levels of other NMDA receptor subunits. Our STHdh^Q7/Q7^ cells were sensitive to NMDA in a dose-dependent manner (Supplementary Figure S2). From these dose-dependency assays, which were similar to those previously reported by Xifro *et al.*,^26^ we selected the standard dose of 1 mM NMDA for further experiments. THC and HU-210 rescued cells from 1-mM NMDA induced death, and blockade of PI3K (with wortmannin), Akt (with Akti-1/2) or mTORC1 (with rapamycin) abrogated cannabinoid-evoked cytoprotection (Figure 1b).

As BDNF plays a key protective role on MSNs, and an association between BDNF expression and CB_1_ receptor function occurs in several pathophysiological settings,^5, 7^ we examined the possible involvement of BDNF in cannabinoid-induced neuroprotection. K252a, an inhibitor of the tyrosine kinase activity of the BDNF receptor TrkB, abrogated THC- and HU-210-induced neuroprotection (Figure 1c). A similar preventive effect was observed when *BDNF* or *TrkB* expression was silenced with specific siRNAs (which diminished total *BDNF* or *TrkB* mRNA levels to 29±10% or 47±10% of control siRNA-transfected cells, respectively; *n*=4–6 experiments, *P*<0.01; Figure 1d). Likewise, the involvement of the PI3K/Akt/mTORC1/BDNF axis in CB_1_ receptor-evoked neuroprotection was also evident (1) when quinolinic acid instead of NMDA was used as excitotoxin (Supplementary Figure S3), and (2) when primary mouse striatal neurons (Ct values of NMDA receptor subunits: NR1, 28; NR2A, 31; NR2B, 26; NR2C, 32; and NR2D, 30) instead of STHdh^Q7/Q7^ cells were used as cellular model (Figure 1e). Specifically, the protective effect of cannabinoids in those two experimental systems was prevented by the CB_1_-selective antagonist SR141716 (rimonabant) or upon blockade of the PI3K/Akt/mTORC1/BDNF pathway (Supplementary Figure S3 and Figure 1e).

### The CB_1_ receptor induces *BDNF* promoter IV via PI3K/Akt/mTORC1

**Figure 2:**
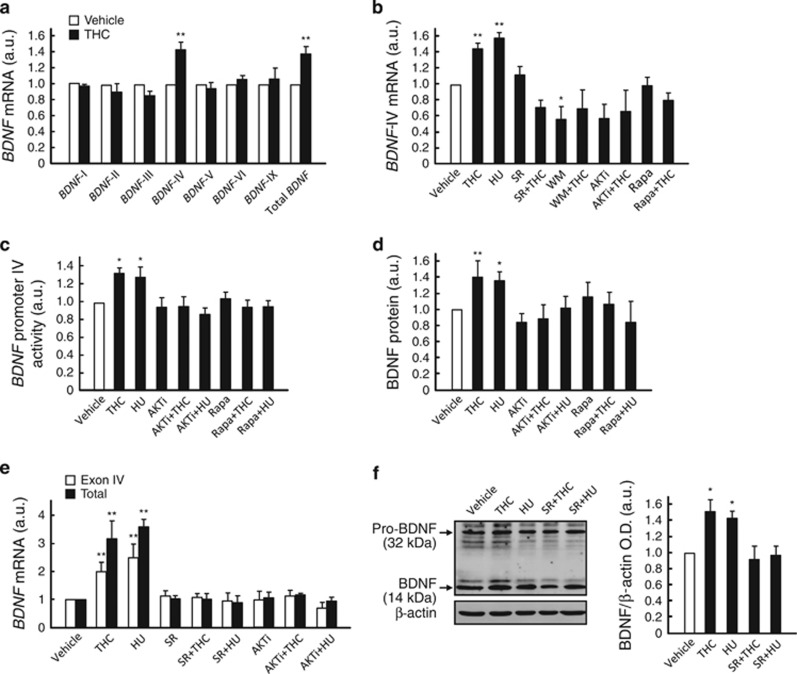
The CB_1_ receptor induces *BDNF* promoter IV via PI3K/Akt/mTORC1. (**a**) STHdh^Q7/Q7^ cells were incubated for 15 h with vehicle or 0.5-*μ*M THC and the levels of *BDNF* transcripts with the indicated 5′ exons were determined (*n*=3–4 experiments). (**b**) STHdh^Q7/Q7^ cells were incubated for 15 h with vehicle or 0.5-*μ*M THC, 10-nM HU-210, 0.25-*μ*M SR141716, 0.2-*μ*M wortmannin, 0.1-*μ*M Akti-1/2 and/or 30-nM rapamycin, and the levels of *BDNF* transcripts containing exon IV were determined (*n*=3–4 experiments). (**c**) STHdh^Q7/Q7^ cells were transfected with a construct harboring a 0.5-kb human *BDNF* promoter IV fused to the luciferase reporter gene and subsequently incubated for 15 h with 0.5-*μ*M THC, 10-nM HU-210, 0.1-*μ*M Akti-1/2 and/or 30-nM rapamycin. Relative promoter activity is shown (*n*=4–6 experiments). (**d**) STHdh^Q7/Q7^ cells were incubated for 24 h with vehicle or 0.5-*μ*M THC,10-nM HU-210, 0.1-*μ*M Akti-1/2 and/or 30-nM rapamycin, and the levels of BDNF protein were determined (*n*=3–4 experiments). (**e**, **f**) Primary mouse striatal neurons were incubated with vehicle or 0.3-*μ*M THC, 10-nM HU-210, 0.25-*μ*M SR141716 and/or 0.1-*μ*M Akti-1/2, and the levels of exon IV-containing and total *BDNF* transcripts (**e**; qPCR; 15-h incubation with the additions; *n*=3–4 experiments) as well as of BDNF protein were determined (**f**; western blot; 24-h incubation with the additions; *n*=4 experiments; quantification of mean±S.E.M. optical density (O.D.) values relative to those of *β*-actin as well as a representative blot are shown). Data were analyzed using ANOVA with *post hoc* Student-Newman-Keuls test. *P<0.05 and **P<0.01 from the corresponding vehicle-treated cells

The *BDNF* gene consists of multiple promoters and 5′ untranslated exons, together with a common 3′ protein-coding exon. After transcription and splicing, one of the 5′ exons is joined to the single coding exon, therefore resulting in different *BDNF* mRNA forms but an identical BDNF protein.^28, 29^ To obtain direct evidence for the CB_1_ receptor-mediated control of BDNF expression in STHdh^Q7/Q7^ cells, we evaluated the effect of cannabinoids on the best characterized *Bdnf* gene promoters by using exon-specific qPCR primers. THC upregulated total *BDNF* transcripts (Ct=23) and, specifically, exon IV-containing *BDNF* transcripts (Ct=27; Figure 2a). Hence, *Bdnf* promoter IV was subsequently studied in further detail. THC-induced accumulation of exon IV-containing transcripts was mimicked by HU-210 and prevented by SR141716 (Figure 2b). As for cannabinoid-evoked neuroprotection (see above), blockade of the PI3K/Akt/mTORC1 pathway prevented the cannabinoid-induced increase of exon IV-containing transcripts (Figure 2b).

We next used additional approaches to substantiate a CB_1_ receptor-induced activation of *BDNF* promoter IV. (1) We transfected STHdh^Q7/Q7^ cells with a construct that contains a human *BDNF* promoter IV fused to the luciferase reporter gene,^30^ and found that promoter IV activity was enhanced by THC and HU-210, this effect being abrogated by blockade of Akt or mTORC1 (Figure 2c). (2) BDNF protein levels, as determined by ELISA in STHdh^Q7/Q7^ cell-culture extracts, were also increased by THC and HU-210 in an Akt- and mTORC1-dependent manner (Figure 2d). (3) We isolated primary mouse striatal neurons and found that CB_1_ receptor agonism increased both exon IV-containing (Ct=29) and total *BDNF* transcripts (Ct=27), as determined by qPCR (Figure 2e), as well as BDNF protein levels, as determined by western blot (Figure 2f; we were unable to reliably quantify BDNF by ELISA in neuron-culture supernatants). These neuron cultures had only ~5% (*n*=3 cultures) of contaminating glial fibrillary acidic protein (GFAP)-positive cells (assumed to be astroglia), and their content in BDNF/GFAP-double-positive cells relative to total BDNF-positive cells was negligible (Supplementary Figure S4). (4) We prepared mouse-brain organotypic cultures and found that THC increased striatal BDNF protein expression, as evidenced by western blot and immunofluorescence (Supplementary Figure S5).

### Multiple transcription factors are involved in the CB_1_ receptor-mediated induction of *BDNF* promoter IV

**Figure 3:**
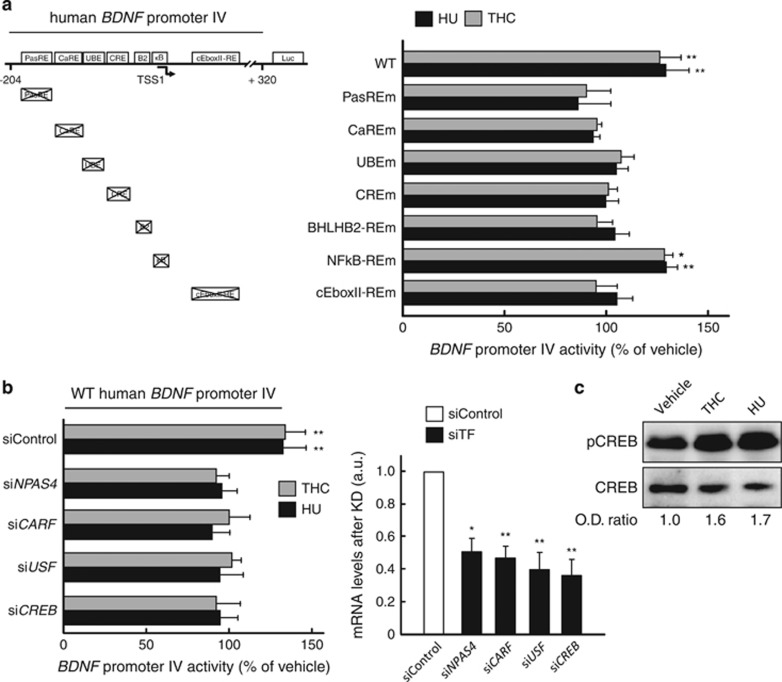
Multiple transcription factors are involved in the CB_1_ receptor-mediated induction of *BDNF* promoter IV. (**a**) STHdh^Q7/Q7^ cells were transfected with a construct harboring a WT 0.5-kb human *BDNF* promoter IV fused to the luciferase reporter gene or with the same construct containing mutations (m) in the indicated response elements (RE). Cells were subsequently incubated for 15 h with vehicle, 0.5-*μ*M THC or 10-nM HU-210. Promoter activity relative to vehicle incubations is shown (*n*=4–6 experiments). (**b**) STHdh^Q7/Q7^ cells were transfected with a non-targeted siRNA or siRNAs directed against the indicated transcription factors. After 24 h, they were transfected with the aforementioned WT *BDNF* promoter IV reporter construct, and, after an additional 24-h period, cells were incubated with vehicle, 0.5-*μ*M THC or 10-nM HU-210 for 15 h. Left: promoter activity relative to vehicle incubations; right: knock-down (KD) efficacy of the siRNAs directed against the corresponding transcription factor (siTF; *n*=4–6 experiments). Data were analyzed using unpaired Student's *t*-test. *P<0.05 and **P<0.01 from the corresponding vehicle-treated cells or siControl-transfected cells (**b**, left). (**c**) STHdh^Q7/Q7^ cells were incubated for 30 min with vehicle, 0.5-*μ*M THC or 10-nM HU-210, lysed and used for western blot analyses. Quantification of mean optical density (O.D.) values of pCREB relative to those of total CREB as well as a representative blot are shown (*n*=3–4 experiments)

We next aimed at characterizing the specific regions of *BDNF* promoter IV involved in the CB_1_ receptor-dependent control of gene transcription. For this purpose, STHdh^Q7/Q7^ cells were transfected with *BDNF* promoter IV-luciferase reporter constructs containing mutations in *cis*-elements that control neuronal *BDNF* promoter IV upon different stimuli.^30, 31^ The cannabinoid-evoked activation of wild-type *BDNF* promoter IV was not evident when mutations were introduced in (1) bHLH-PAS transcription factor-response element (PasRE), to which neuronal PAS domain protein 4 (NPAS4)-aryl hydrocarbon receptor nuclear translocator 2 (ARNT2) dimers bind; (2) Ca^2+^-response element 1 (CaRE1), to which calcium-responsive transcription factor (CaRF) binds; (3) upstream stimulatory factor-binding element (UBE), to which upstream stimulatory factors (USFs) bind; (4) cAMP/Ca^2+^-response element (CRE), to which cAMP response element-binding protein (CREB) binds; (5) basic helix-loop-helix B2 (BHLHB2)-response element (BHLHB2-RE); and (6) conserved E-box element 2 (cEbox2; Figure 3a). Cannabinoid action on *BDNF* promoter IV was not affected when the NF*κ*B-response element (NF*κ*B-RE) was mutated (Figure 3a).

To evaluate the involvement of the best-defined transcription factors that bind to the aforementioned *BDNF* promoter IV regulatory elements, we silenced the expression of NPAS4, CaRF, USF and CREB with specific siRNAs and measured cannabinoid-evoked activation of the wild-type *BDNF* promoter IV. Knocking-down the expression of any of these transcription factors abrogated the cannabinoid-induced activation of *BDNF* promoter IV (Figure 3b), thus suggesting that all of them are necessary for the latter process to occur. Accordingly, CREB phosphorylation in its critical activatory S133 residue was enhanced by cannabinoid challenge (Figure 3c).

### CB_1_ receptor antagonism attenuates BDNF upregulation induced by pharmacogenetic activation of striatal neurons

To evaluate the relevance of the CB_1_ receptor in the physiological control of striatal BDNF expression, we selectively manipulated MSN activity by the *designer receptor exclusively activated by designer drug* (DREADD) pharmacogenetic technique. This tool is based on the molecular evolution of muscarinic acetylcholine receptors, leading to a G_q_ protein-coupled receptor with negligible affinity for the native agonist (acetylcholine) but to which the pharmacologically inert agonist clozapine-*N*-oxide (CNO) binds with high potency and efficacy,^32^ thus allowing the remote control of neuronal activity in specific cell populations *in vivo*.^33^

**Figure 4:**
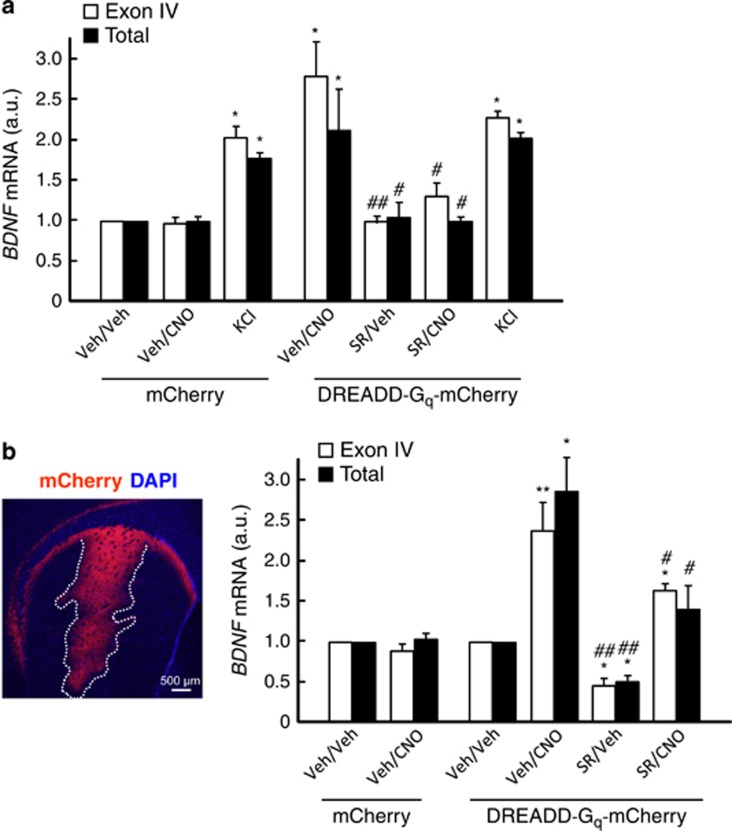
CB_1_ receptor antagonism attenuates BDNF upregulation induced by pharmacogenetic activation of striatal neurons. (**a**) STHdh^Q7/Q7^ cells were nucleofected with a construct expressing hM3Dq-mCherry (or mCherry alone as control). Two days after, cells were treated with 50-*μ*M CNO (or H_2_O as vehicle) plus 0.25-*μ*M SR141716 (or 0.1% DMSO as vehicle) for 4 h, and the levels of exon IV-containing and total *BDNF* transcripts were determined by qPCR (*n*=3–4 experiments). KCl (25 mM) was used as a control of activity-dependent *BDNF* expression upregulation. (**b**) Eight-week-old C57BL/6J mice were injected stereotactically into the dorsolateral striatum with a recombinant adeno-associated virus encoding hM3Dq-mCherry (or only mCherry as control) under the control of the CaMKII*α* promoter (*n*=6–8 animals per group). Left: Example of a mouse brain injected with the mCherry construct. The site of infection in the striatum is outlined. Right: Levels of exon IV-containing and total *BDNF* transcripts in the dorsolateral striatum as determined by qPCR 4 h after i.p. injection of CNO (10 mg/kg body weight) with or without SR141716 (1 mg/kg body weight) or the corresponding vehicles. Data were analyzed using unpaired Student's *t*-test. *P<0.05 and **P<0.01 from the corresponding Veh/Veh group; ^#^*P*<0.05 and ^##^*P*<0.01 from the corresponding Veh/CNO group

First, we validated this experimental approach *in vitro*. STHdh^Q7/Q7^ cells were nucleofected with a plasmid encoding a DREADD-G_q_ (hM3Dq) fused to mCherry (or only mCherry) and subsequently treated with CNO (or vehicle). CNO-induced activation of hM3Dq led to an accumulation of both exon IV-containing and total *BDNF* transcripts, and SR141716 prevented this effect (Figure 4a). Next, we injected stereotactically C57BL/6J mice with a recombinant adeno-associated viral vector encoding hM3Dq-mCherry (or only mCherry) into the dorsolateral (motor) striatum (Figure 4b). The expression of the transgene was driven by the calcium/calmodulin-dependent protein kinase II-*α* (CaMKII*α*) promoter in order to confine it to MSNs and avoid other cell populations (e.g., interneurons and glia). Animals were subsequently treated with CNO (or vehicle) in conditions that evoke neuronal activation (one injection of CNO at 10 mg/kg body weight).^34^ This procedure triggered the expression of exon IV-containing and total *BDNF* transcripts in the striatum *in situ*, and, of note, treatment with SR141716 (one injection at 1 mg/kg body weight) produced *per se* the opposite effect to CNO and attenuated the CNO-triggered upregulation of *BDNF* expression (Figure 4b).

### Pathophysiological relevance of CB_1_ receptor-mediated striatal BDNF upregulation in HD

To assess the functional impact of the CB_1_/BDNF axis in a neurodegenerative-disease context *in vivo*, we used the R6/2 mouse, a well-established model of HD.^35^ This devastating disease constitutes so far the best paradigm to study the neuroprotective role of the CB_1_ receptor as this receptor is highly expressed in the basal ganglia by MSNs, the cells that constitute ~90% of total striatal neurons and primarily degenerate in HD, and plays a key role in the control of motor behavior, one of the processes that is typically affected in HD.^4, 12^ In addition, an early and remarkable downregulation of CB_1_ receptor expression has been documented as one of the most characteristic neurochemical alterations of MSNs in HD animal models^36, 37, 38^ and HD patients.^39, 40^ Moreover, we^19^ and others^41^ have provided genetic evidence for a neuroprotective role of the CB_1_ receptor in HD mouse models.

**Figure 5:**
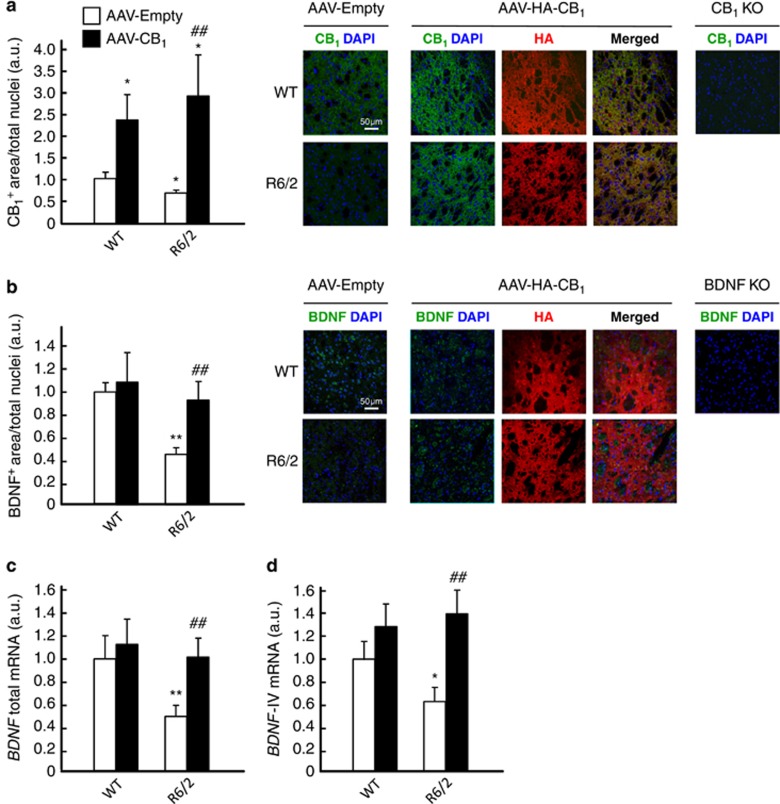
Enforced re-expression of the CB_1_ receptor rescues BDNF expression in the striata of R6/2 mice. R6/2 mice (3.5–4 weeks old) and WT littermates were injected stereotactically into the dorsolateral striatum with a recombinant adeno-associated virus (AAV) encoding HA-tagged CB_1_ receptor or the empty vector as control (*n*=10–12 animals per group). At week 8 of age, animals were killed for histological and qPCR analyses in the striatum. (**a**) CB_1_ receptor immunoreactivity (relative values of CB_1_ receptor-positive area/total cell nuclei). Slices from the dorsolateral striatum of *CB*_*1*_^−/−^ mice^70^ were used as control of staining specificity. Representative images are shown. (**b**) BDNF immunoreactivity (relative values of BDNF-positive area/total cell nuclei). Slices from the dorsolateral striatum of neuron-specific, neurofilament L-conditional BDNF knockout (*Bdnf*^*floxed/-;Nefl-Cre/+*^) mice (generated at Michael Sendtner's laboratory, University of Würzburg, Germany) were used as control of staining specificity. Representative images are shown. (**c**) Levels of total *BDNF* transcripts. (**d**) Levels of exon IV-containing *BDNF* transcripts. Data were analyzed using unpaired Student's *t*-test. *P<0.05 and **P<0.01 from the corresponding WT-empty group; ^##^*P*<0.01 from the corresponding R6/2-empty group

**Figure 6:**
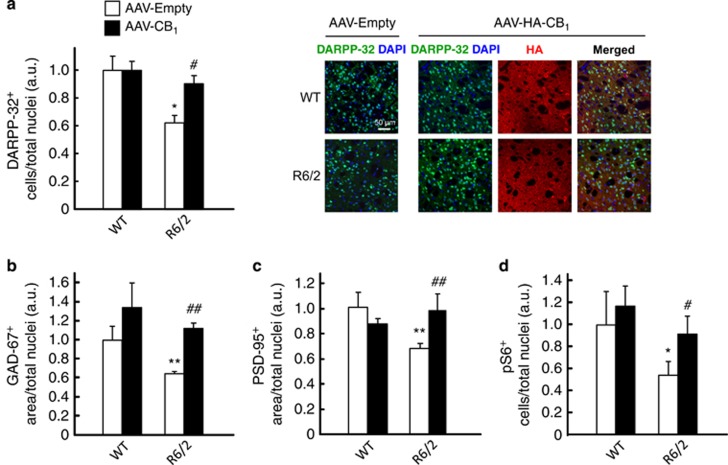
Enforced re-expression of the CB_1_ receptor rescues HD-like molecular-pathology markers in the striata of R6/2 mice. R6/2 mice (3.5–4 weeks old) and WT littermates were injected stereotactically into the dorsolateral striatum with a recombinant adeno-associated virus (AAV) encoding HA-tagged CB_1_ receptor or the empty vector as control (*n*=10–12 animals per group). At week 8 of age, animals were killed for histological analyses in the striatum. (**a**) DARPP-32 immunoreactivity (relative values of DARPP-32-positive cells/total cell nuclei). Representative images are shown. (**b**) GAD-67 immunoreactivity (relative values of GAD-67-positive area/total cell nuclei). (**c**) PSD-95 immunoreactivity (relative values of PSD-95-positive area/total cell nuclei). (**d**) Phospho-S6 immunoreactivity (relative values of pS6-positive cells/total cell nuclei). Data were analyzed using unpaired Student's *t*-test. *P<0.05 and **P<0.01 from the corresponding WT-empty group; ^#^*P*<0.05 and ^##^*P*<0.01 from the corresponding R6/2-empty group

**Figure 7:**
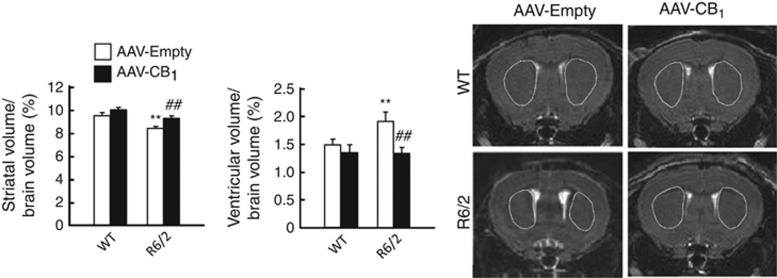
Enforced re-expression of the CB_1_ receptor rescues HD-like striatal atrophy in R6/2 mice. R6/2 mice (3.5–4 weeks old) and WT littermates were injected stereotactically into the dorsolateral striatum with a recombinant adeno-associated virus (AAV) encoding HA-tagged CB_1_ receptor or empty vector as control (*n*=10–12 animals per group). The volume of the striatum and lateral ventricles relative to total brain volume of 8-week-old animals is represented. Representative MRI pictures are shown. Striata are outlined. Data were analyzed using unpaired Student's *t*-test. **P<0.01 from the corresponding WT-empty group; ^##^*P*<0.01 from the corresponding R6/2-empty group

To test whether this neuroprotective effect relies on BDNF signaling, we injected sterotactically 3.5- to 4-week-old R6/2 mice (or wild-type littermates) with a recombinant adeno-associated viral vector encoding CB_1_ receptor (or empty vector) into the dorsolateral striatum. This procedure allowed the subsequent re-expression of the CB_1_ receptor (Figure 5a) as well as BDNF protein (Figure 5b), total *BDNF* mRNA (Figure 5c; Ct=26) and exon IV-containing *BDNF* mRNA (Figure 5d; Ct=29). Reactivation of the CB_1_/BDNF axis also normalized the HD-like molecular-pathology profile of these animals, as determined by the recovered levels of the MSN marker dopamine- and cAMP-regulated phosphoprotein of 32 KDa (DARPP-32; Figure 6a; confirmation by stereological counting shown in Supplementary Figure S6), the *pan*-GABAergic-neuron marker glutamic acid decarboxylase 67 kDa isoform (GAD-67; Figure 6b), the post-synaptic marker post-synaptic density protein 95 (PSD-95; Figure 6c) and the mTORC1-activity marker phosphorylated (activated) ribosomal S6 protein (Figure 6d). In addition, CB_1_ receptor re-expression rescued striatal atrophy, the main neuropathological hallmark of HD, as determined by MRI analysis (Figure 7). Cortical and hippocampal volumes, used as controls, were not significantly different in 8-week-old wild-type and R6/2 mice injected with CB_1_ receptor-encoding or empty viral vectors (Supplementary Figure S7). (We note that overexpression of the CB_1_ receptor in the striata of wild-type mice did not lead to a significant upregulation of BDNF or other markers of neuronal integrity/functionality. As MSNs express very large amounts of CB_1_ receptors, it is conceivable that the CB_1_ receptor-response system would be essentially saturated in the normal setting but not in conditions of restricted receptor function such as HD. In this regard, agonist-stimulated [^35^S]GTP*γ*S-binding studies have shown that when higher expression levels of CB_1_ receptors occur, for example, in the striatum *versus* other brain regions,^42^ in GABAergic *versus* glutamatergic terminals^43^ or in CB_1_^+/+^ *versus* CB_1_^+/−^ mice,^44^ the receptors couple with little efficacy to G proteins, thus making signaling in CB_1_ highly expressing systems refractory to stimulation by mere increases in total receptor numbers.)

Finally, we analyzed a series of *post-mortem* human caudate-putamen samples for concerted changes in CB_1_ receptor and BDNF immunoreactivity. In line with Zuccato and Cattaneo,^17^ we found a significant reduction of CB_1_ receptor-positive, BDNF-positive and CB_1_/BDNF-double-positive neurons in HD patients compared to control subjects (Supplementary Figure S8a). Western blot analyses, which had previously shown a decrease in CB_1_ receptor protein expression in caudate-putamen specimens of HD patients,^19^ evidenced a parallel reduction of BDNF protein expression in those samples (Supplementary Figure S8b). This was associated to a concomitant decrease in the levels of pCREB (Supplementary Figure S8b), a key signaling marker of the CB_1_/BDNF axis described above. These findings therefore suggest that a functional link between the CB_1_ receptor and BDNF could also occur in the human brain.

## Discussion

Here we show that, in the mouse striatum, CB_1_ receptor engagement upregulates BDNF expression, through which it can confer neuroprotection against excitotoxicity *in vitro* and mutant huntingtin-induced toxicity *in vivo*. On mechanistic grounds, this CB_1_ receptor-mediated induction of *BDNF* gene expression relies on the activation of the PI3K/Akt/mTORC1 pathway, which, in turn, targets *BDNF* promoter IV, a promoter that is also responsive to various types of neuronal activity-related stimuli in the mouse, rat and human *BDNF* gene.^10, 29, 30^ The induction of *BDNF* promoter IV evoked by the CB_1_ receptor-mediated activation of the PI3K/Akt/mTORC1 pathway appears to be a complex process, as several responsive elements and transcription factors are involved. The observation that CREB is necessary for the CB_1_ receptor-mediated induction of *BDNF* promoter IV fits with the pivotal role of this transcription factor in the regulation of BDNF action.^10, 45^ In fact, mice with a specific knock-in mutation in the CRE of *Bdnf* promoter IV display impaired sensory experience-induced expression of BDNF and defective development of cortical inhibitory circuits.^46^ It is thus conceivable that the rapid and pleiotropic triggering of Ca^2+^-, cAMP-, ERK- and/or Akt-related signals will converge in the immediate/early activation of CREB and CaRF, which, by binding to CRE^30^ and CaRE1,^31, 47^ respectively, could initiate *BDNF* promoter IV transcription. Of note, and in line with our data, the Ca^2+^ influx-dependent early activation of CRE/CaRE3 (via CREB) and CaRE1 (via CaRF) in the rat *BDNF* promoter IV is abrogated by genetic or pharmacological blockade of PI3K signaling.^31^ Upon CREB and CaRF activation, at later time points other transcription factors, such as NPAS4 via PasRE,^30, 48^ could be important for sustaining the transcriptional activity of *BDNF* promoter IV under different conditions, including CB_1_ receptor activation, as reported here.

The relation between CB_1_ receptor activation and BDNF expression appears to be a region-specific process. Thus, this association has been clearly established in the mouse hippocampus by experiments involving CB_1_ receptor gain of function (CB_1_ receptor pharmacological agonism) and loss of function (CB_1_ receptor genetic inactivation, CB_1_ receptor pharmacological antagonism) conducted in various *in vitro* (tissue slices, cell cultures) and *in vivo* (whole mice) experimental systems.^8, 49, 50, 51, 52, 53, 54^ In line with our present study in the mouse dorsolateral striatum, THC administration increased BDNF expression in the rat ventral striatum.^55^ However, and in striking contrast with the hippocampus and the striatum, BDNF expression in the mouse cortex, which expresses high levels of the CB_1_ receptor,^1^ was unaffected by either THC administration^19, 51^ or CB_1_ receptor genetic ablation,^19, 52^ while another study found only very marginal increases in BDNF levels in the medial prefrontal cortex and the frontoparietal cortex upon THC injection to rats.^55^ Hence, albeit for hitherto unknown molecular reasons, BDNF expression seems to be much more refractory to CB_1_ receptor activation in the cortex than in the striatum or hippocampus.

This zonation of the CB_1_/BDNF axis in the brain is certainly relevant in the context of our findings because MSNs are known to receive BDNF from the cortex via the well-established corticostriatal pathway.^56^ In addition, significant amounts of *BDNF* mRNA have been found in the striatum, thus indicating that striatal BDNF can also be produced *in situ*.^19, 29, 57, 58^ Moreover, our DREADD experiments, by allowing the remote and selective control of MSN activity, provide robust evidence for the activity-dependent production of BDNF in mouse MSNs *in vivo*. Further support to this notion comes from the findings that mutant huntingtin affects axonal transport of BDNF in striatal neurons but not in cortical neurons,^59^ and that dopamine receptor heteromers control BDNF production by striatal neurons *in situ*.^60^ All these observations do not downplay the corticostriatal pathway as a key source of BDNF for MSNs in the normal brain. For example, in our hands, significant amounts of *BDNF* transcripts are readily detected in the adult-mouse striatum, but their levels are lower than those found in the cortex (mRNA levels in the striatum relative to the cortex: total *BDNF*, 24±2% *BDNF*-IV, 20±4% *n*=8 animals). However, it is likely that, under particular pathophysiological situations, BDNF production can increase in the striatum *in situ*, thereby complementing the bulk supply of BDNF from the cortex with a local –and thus spatially privileged- extra source of the neurotrophin for MSNs. The multiple lines of evidence provided by this study, together with the aforementioned lack of effect of CB_1_ receptor activation on cortical BDNF expression, strongly support that the CB_1_ receptor-mediated upregulation of striatal BDNF is a striatum-autonomous effect rather than the consequence of an enhanced anterograde supply of BDNF from the cortex. Nonetheless, the CB_1_/BDNF connection in MSNs can be more complex and be accompanied, for example, by a reciprocal BDNF-dependent control of CB_1_ receptor function.^61^

A key unanswered question in many neurodegenerative diseases is what precise factors dictate the selective damage of a particular neuronal population. Regarding HD, the disease has long been known to be caused by an expanded polyglutamine tract in the *N*-terminal domain of the huntingtin protein,^62^ but the mechanisms by which MSNs are highly vulnerable to mutant huntingtin are still incompletely understood. We^19^ and others^41^ have provided genetic evidence for a neuroprotective role of the CB_1_ receptor in two transgenic models of HD, which could open similar studies on other neurodegenerative diseases, such as Alzheimer's disease,^63, 64, 65^ in which CB_1_ receptor levels are known to be downregulated during disease state. Unfortunately, the precise relevance of CB_1_ receptor and BDNF downregulation in HD pathology are not completely understood. For example, regarding the latter issue, Plotkin *et al.*^66^ have recently shown that, although reduced BDNF availability in the striatum may contribute to HD pathology,^17^ a major pathogenic mechanism seems to rely on an aberrant BDNF signaling via p75 neurotrophin receptors located on indirect-pathway MSNs, which adds to the previously reported alterations of BDNF signaling via TrkB.^21, 22^ These possibilities notwithstanding, here we cogently show that, in the striatum of the R6/2 mouse *in vivo*, changes in CB_1_ receptor expression parallel changes in the expression of BDNF and key markers of disease neuropathology, thus supporting the notion that BDNF may be a *bona fide* marker not only of HD neurodegeneration^17^ but also of CB_1_ receptor-evoked neuroprotection.

## Materials and Methods

### Animals

Hemizygous male mice transgenic for exon 1 of the human huntingtin gene with a greatly expanded CAG tract (R6/2 mice; 155–175 CAG repeats)^35^ and wild-type littermates were purchased from The Jackson Laboratory (Bar Harbor, ME, USA). C57BL/6J mice (Harlan, Barcelona, Spain) were used to obtain organotypic and cell cultures, as well as to conduct DREADD experiments. Animals were maintained as described.^19^ All animal handling procedures were approved by the Complutense University Animal Research Committee in accordance with the directives of the European Commission.

### Cell cultures

Conditionally, immortalized mouse striatal neuroblasts expressing wild-type huntingtin and infected with a defective retrovirus transducing the temperature-sensitive A58/U19 large T antigen (designated as STHdh^Q7/Q7^ cells),^24^ were grown at 33°C in DMEM supplemented with 10% fetal bovine serum, 1 mM sodium pyruvate, 2 mM l-glutamine and 400 *μ*g/ml geneticin.

Primary striatal neurons were obtained from 2-day-old C57BL/6J mice using a papain dissociation system (Worthington, NJ, USA). Striata were dissected and cells were seeded on plates pre-coated with 0.1 mg/ml poly-d-lysine at 200 000 cells/cm^2^ in the Neurobasal medium supplemented with B27 and GlutaMax (Gibco, Carlsbad, CA, USA).

### Cell viability

STHdh^Q7/Q7^ cells were transferred to a serum-free medium for 24 h and incubated for a further 5 h in home-made Locke's solution (154-mM NaCl, 5.6-mM KCl, 2.3-mM CaCl_2_, 3.6-mM NaHCO_3_, 5-mM HEPES (Lonza, Verviers, Belgium), 20-mM glucose and 10-*μ*M glycine), supplemented or not with NMDA, together with the cannabinoid receptor agonists THC (The Health Concept, Richelbach, Germany) or HU-210 (Tocris, Bristol, UK), the PI3K inhibitor wortmannin (Sigma-Aldrich, Barcelona, Spain), the Akt inhibitor Akti-1/2 (Calbiochem, San Diego, CA, USA), the mTORC1 inhibitor rapamycin (Tecoland, Irvine, CA, USA), the tyrosine kinase inhibitor K252a (Calbiochem) or the respective vehicle (DMSO, 0.1–0.2% (v/v) final concentration). The medium was subsequently replaced by NMDA/serum-free DMEM supplemented with the corresponding drugs, and cell viability was determined after 24 h by the 3-(4,5-dimethylthiazol-2-yl)-2,5-diphenyltetrazolium bromide (MTT) test.

Another set of STHdh^Q7/Q7^ cell-viability experiments was conducted with quinolinic acid (Sigma-Aldrich) as neurotoxic agent. STHdh^Q7/Q7^ cells were transferred to serum-free DMEM for 24 h and subsequently incubated in this medium, supplemented or not with quinolinic acid, together with THC, HU-210, the CB_1_-selective antagonist SR141716 (rimonabant; kindly given by Sanofi-Aventis, Montpellier, France), Akti-1/2, rapamycin and K252a, or the respective vehicle (DMSO, 0.1–0.2% (v/v) final concentration) for 24 h. Cell viability was determined by the MTT test.

Primary C57/BL6J-mouse striatal neurons, grown for 2 days *in vitro*, were incubated for 30 min in the aforementioned Locke's solution, supplemented or not with NMDA, together with THC, HU-210, SR141716, Akti-1/2, rapamycin and K252a, or the respective vehicle (DMSO, 0.1–0.2% (v/v) final concentration). The medium was subsequently replaced by NMDA-free Neurobasal medium supplemented with B27 and GlutaMax (Gibco), plus the corresponding drugs, and cell viability was determined after 2 h by the MTT test.

### Cell transfection/nucleofection

Cells were transfected transiently with an ON-TARGETplus SMARTpool m*Bdnf* siRNA (Re. L-042566-00) or m*TrkB* (= m*Ntrk2*) siRNA (Re. L-048017-00), or a non-targeted SMARTpool siRNA (Re. D-001810-10), using the DharmaFECT 1 transfection reagent (Thermo Fisher, Lafayette, CO, USA).

For the luciferase reporter assays, cells were transfected transiently with constructs expressing a 524-bp human *BDNF* promoter IV cloned into pGL4.15 (luc2P/Hygro), or the same promoter with mutated PasRE, CaRE, UBE, CRE, BHLHB2-RE, NFκB-RE or cEbox II sites also cloned into pGL4.15 (luc2P/Hygro), as described,^30^ using Lipofectamine 2000 (Invitrogen, Madrid, Spain). For normalization, a renilla/pGL4.83 construct (driven by the thymidine kinase promoter) was cotransfected. Luciferase assays were performed with the Dual-Glo luciferase assay system (Promega Biotech Ibérica, Barcelona, Spain).

Other promoter activity experiments were conducted in cells transfected transiently with ON-TARGETplus SMARTpool m*Npas4* (Re. L-054722-01), m*Carf* (Re. L-051736-01), m*Usf1* (Ref. L-040656-00) or m*Creb1* (Re. L-040959-01) siRNAs, or the aforementioned non-targeted SMARTpool siRNA, using the DharmaFECT 1 transfection reagent (Thermo Fisher).

For DREADD experiments *in vitro*, cells were nucleofected with a construct expressing the hM3Dq receptor fused to mCherry (kindly provided by Bryan L Roth, University of North Carolina, Chapel Hill, NC, USA),^34^ or mCherry alone as control vector, under the CAG promoter, by using an Amaxa mouse-neuron nucleofector kit (Lonza, Madrid, Spain). Cells were treated in the serum-free medium, 2 days after nucleofection, with CNO (or H_2_O as vehicle) plus SR141716 (or 0.1% DMSO as vehicle).

### Organotypic cultures

Corticostriatal slices (300-*μ*m thick) were obtained from adult (8 weeks old) C57/BL6J mice, and cultured under semidry conditions in the Neurobasal medium supplemented with B27 (1%), N2 (1%), glutamine (1%), penicillin/streptomycin (1%), Fungizone (1% Gibco) and ciprofloxacin (5 *μ*g/ml), as described.^19^ Slices were incubated for 24 h with THC or vehicle (DMSO, 0.1% (v/v) final concentration) and subsequently fixed with formalin and processed in 15-*μ*m sections, which were analyzed at equivalent regions of the rostral to caudal axis. Counting of BDNF and NeuN immunoreactivity (see below) was conducted in the dorsolateral striatum in a 1-in-6 series per slice.

### Real-time PCR

RNA was isolated using TRIzol reagent or RNeasy (Invitrogen, Carlsbad, CA, USA). cDNA was obtained using Transcriptor (Roche, Basel, Switzerland). Real-time PCR (qPCR) assays were performed using the FastStart SYBR Green Master (Roche) and probes were obtained from the Universal Probe Library Set (Roche). The mRNA levels of the different *BDNF* exons were determined with previously described primers.^29, 30^ Other primers used are shown in Supplementary Table SI. Amplifications were run in a 7900 HT-Fast Real-time PCR system (Applied Biosystems, Foster City, CA, USA). Relative gene expression data were determined by the 2^−ΔΔCt^ method. Each value was adjusted to *β*-actin levels as reference.

### Viral vectors

HA-tagged rat CB_1_ cannabinoid receptor was subcloned in a rAAV expression vector with a CAG promoter by using standard molecular cloning techniques. Vectors were of an AAV1/AAV2-mixed serotype, and were generated by calcium phosphate transfection of HEK293T cells and subsequent purification as described.^67, 68^ R6/2 mice (3.5–4 weeks old) and their wild-type littermates were injected stereotactically with the viral vectors (in 1.5 *μ*l PBS) into the dorsolateral striatum. Each animal received one bilateral injection at coordinates (to bregma): anteroposterior −0.5, lateral ±1.4, dorso-ventral −2.7. MRI analyses were conducted at 8 weeks of age. Mice were subsequently killed by intracardial perfusion and their brains were excised for immunofluorescence and qPCR analyses.

For DREADD experiments *in vivo*, 8-week-old male C57BL/6J animals were injected stereotactically with viral vectors expressing the hM3Dq receptor fused to mCherry (or mCherry as control) under a minimal CaMKII*α* promoter into the dorsolateral striatum as described before.^9^ Six weeks after surgery, animals were injected with SR141716 (1 mg/kg body weight, i.p.) or vehicle (1% (v/v) DMSO in Tween 20 (Panreac, Barcelona, Spain)/saline (1 : 18, v/v)) and, 10 min later, with CNO (10 mg/kg body weight, i.p.) or vehicle (PBS). Animals were killed 4 h after the CNO injection, and their dorsolateral striata were quickly dissected on ice and frozen at −80°C for subsequent analyses.

### Western blot

Western blot analysis was conducted with antibodies raised against phosphorylated Akt (1 : 1000; Cell Signaling, Danvers, MA, USA), total Akt (1 : 1000; Cell Signaling), phosphorylated S6 ribosomal protein (1 : 1000; Cell Signaling), total S6 ribosomal protein (1 : 1000; Cell Signaling), phosphorylated ERK (1 : 1000; Cell Signaling), total ERK (1 : 1000; Cell Signaling), phosphorylated JNK (1 : 1000; Cell Signaling), total JNK (1 : 1000; Cell Signaling), phosphorylated p38 (1 : 1000; Cell Signaling), total p38 (1 : 1000 Cell Signaling), PKA phosphorylated substrates (1 : 1000; Cell Signaling), phosphorylated CREB (1 : 1000, Cell Signaling), total CREB (1 : 1000, Cell Signaling), BDNF (1 : 1000, Santa Cruz Biotechnology, Santa Cruz, CA, USA), *α*-tubulin (1 : 4000, Sigma-Aldrich) and *β*-actin (1 : 4000, Sigma-Aldrich), following standard procedures. Specifically, samples from cell cultures, organotypic cultures and human *post-mortem* brains (obtained from the Banco de Tejidos para Investigación Neurológica, Madrid, Spain, as previously described)^19^ were lysed in a buffer containing 50-mM Tris, 0.3% CHAPS, 1-mM EDTA, 1-mM EGTA, 50-mM NaF, 10-mM sodium *β*-glycerophosphate, 5-mM sodium pyrophosphate and 1-mM sodium orthovanadate (pH 7.5) supplemented with a protease inhibitor cocktail (Roche, Madrid, Spain), 0.1-mM PMSF and 1-*μ*M microcystin. The running buffer consisted of 200-mM glycine, 25-mM Tris and 0.1% SDS (pH 8.3), and the transfer buffer contained 200-mM glycine, 25-mM Tris and 20% methanol (pH 8.3). Blots were incubated with Tris-buffered saline (20-mM Tris and 0.5-mM NaCl, pH 7.5)/Tween 20 (0.05%) supplemented with 1% bovine serum albumin. Densitometric analysis was performed with Quantity One software (Bio-Rad, Hercules, CA, USA).

### Immunomicroscopy (mouse samples)

Coronal free-floating sections (30 *μ*m-thick) were obtained from paraformaldehyde-perfused mouse brains. Organotipic cultures were obtained as described before. Samples were incubated with antibodies against CB_1_ cannabinoid receptor (1 : 500; Frontier Science, Hokkaido, Japan), HA (1 : 500, Roche), BDNF (1 : 300; generated at Michael Sendtner's laboratory, University of Würzburg, Germany),^19^ DARPP-32 (1 : 1000; BD, Franklin Lakes, NJ, USA), GAD-67 (1 : 250; Chemicon, Temecula, CA, USA), PSD-95 (1 : 1000, Abcam, Cambridge, UK), phosphorylated S6 ribosomal protein (1 : 200; Cell Signaling) or NeuN (1 : 400; Chemicon), followed by staining with the corresponding highly cross-adsorbed Alexa Fluor 488, 594 or 647 antibodies (1 : 500; Molecular Probes, Leyden, The Netherlands).^19^ GFAP was stained with an anti-GFAP-Cy3 antibody (1 : 1000, Sigma-Aldrich). Samples were subsequently incubated with DAPI (1 : 10 000, Roche) for 10 min, washed with PBS and mounted in Mowiol (Calbiochem, Madrid, Spain). Counting of brain sections was conducted in the caudate-putamen area in a 1-in-10 series per animal, ranging from bregma +1.5 mm to −0.5 mm coronal coordinates. Data were calculated as immunoreactive area per total cell nuclei, except for DARPP-32, phospho-S6 and NeuN, in which immunoreactive cells per total cell nuclei were counted. Confocal fluorescence images were acquired using TCS-SP2 software and a SP2 AOBS microscope (Leica, Wetzlar, Germany). Pixel quantification and co-localization were analyzed with ImageJ software (National Institutes of Health, Bethesda, MA, USA).

Stereological counting of the total number of DARPP-32-positive cells in the rAAV-infected region of the mouse dorsolateral striatum (Supplementary Figure S6) was performed in 30-*μ*m-thick sections with the aforementioned DARPP-32/HA/DAPI staining conditions and within the aforementioned coronal coordinates using the optical fractionator method. A 1-in-8 series per animal was analyzed in an Olympus BX61 microscope (Olympus, Tokyo, Japan) with newCAST software (Visiopharm, Horsholm, Denmark). Volumes were calculated by applying the Cavalieri estimator. The frame area was set to 5625 *μ*m^2^ with a sampling interval of 240 *μ*m at the *x* and *y* level, and the optical dissector constituting a 13-*μ*m-thick fraction of the total section thickness. Results are expressed as number of DARPP-32-immunoreactive cells per mm^3^ of rAAV-infected region. Gundersen's coefficient of error was always below 0.1.

### Immunomicroscopy (human samples)

Paraffin-embedded *post-mortem* 4-*μ*m-thick brain sections containing caudate-putamen were provided by the Tissue Bank at Hospital Universitario Fundación Alcorcón (Madrid, Spain), and were obtained and handled following the ethical guidelines of that institution. Samples (four sections per individual) were obtained from HD donors (grades 3–4; *n*=9; age (years old) and sex: 55♂, 47♀, 45♂, 45♂, 51♂, 51♀, 44♂, 77♀ and 49♂) and control subjects with no background of neuropsychiatric disease (*n*=7; age (years old) and sex: 45♂, 62♀, 36♂, 74♂, 79♂, 75♀ and 69♂). For immunofluorescence analyses,^69^ sections were incubated with anti-BDNF antibody (1 : 50; generated at Michael Sendtner's laboratory) and subsequently with Alexa Fluor 546 antibody (Molecular Probes). Samples were then incubated with anti-CB_1_ cannabinoid receptor antibody (1 : 50; Affinity Bioreagents, Golden, CO, USA), followed by incubation with Alexa Fluor 488 antibody (Molecular Probes), and finally incubated with DAPI (1 : 10 000) for 10 min and washed with PBS. Then, sections were treated with 1% Sudan Black (Sigma-Aldrich) in 70% ethanol to quench endogenous autofluorescence and finally mounted in Mowiol.

### ELISA

STHdh^Q7/Q7^ cells were transferred to the serum-free medium and incubated with vehicle (DMSO, 0.1–0.2% (v/v) final concentration) or THC, HU-210, Akti-1/2 and rapamycin for 24 h as described above. BDNF protein levels were determined upon scraping the cell culture well and combining it with its medium by using a BDNF ELISA System (Promega Biotech Ibérica).

### Magnetic resonance imaging

The volume of the striatum, ventricles, cortex (comprising somatosensory and motor areas) and hippocampus was measured by magnetic resonance imaging in a BIOSPEC BMT 47/40 (Bruker, Ettlingen, Germany) operating at 4.7 T as described.^19^

### Statistics

Data are presented as mean±S.E.M. Statistical comparisons were made by ANOVA with *post hoc* Student-Newman-Keuls test or by unpaired Student's *t*-test, as indicated in each figure legend.

## Abbreviations

BDNF: brain-derived neurotrophic factor
CaMKII*α*: calcium/calmodulin-dependent protein kinase II-*α*
CaRF: calcium-responsive transcription factor
CNO: clozapine-*N*-oxide
CREB: cAMP response element-binding protein
Ct: threshold cycle
DARPP-32: dopamine- and cAMP-regulated phosphoprotein of 32 kDa
DREADD: designer receptor exclusively activated by designer drug
ERK: extracellular signal-regulated kinase
GAD-67: glutamic acid decarboxylase 67 kDa isoform
GFAP: glial fibrillary acidic protein
HD: Huntington's disease
JNK: c-Jun *N*-terminal kinase
MSN: medium-sized spiny neuron
mTORC1: mammalian target of rapamycin complex 1
NMDA: *N*-methyl-d-aspartate
NPAS4: neuronal PAS domain protein 4
PI3K: phosphatidylinositol 3-kinase
PKA: cAMP-dependent protein kinase
PSD-95: post-synaptic density protein 95
THC: Δ^9^-tetrahydrocannabinol
USF: upstream stimulatory factor

## Supplementary Material

Supplemntary Table S1
