# Supplementary material for: The CB1 cannabinoid receptor signals striatal neuroprotection via a PI3K/Akt/mTORC1/BDNF pathway
Source: Cell Death Differ. 2015 Feb 20;22(10):1618–29. doi: 10.1038/cdd.2015.11 (PMC4563779; doi:10.1038/cdd.2015.11)
Supplement: Supplemntary Table S1 [file cdd201511x1.pdf]

Gene	Primer (5´-3´)
m*TrkB*	TGGTGCATTCCATTCACTGT
m*Gapdh*	GGGAAGCTCACTGGCATGGCCTTCC
m*Carf*	CCATATGACGGAATCCCATT
m*Creb1*	ACTAGCAGTGGGCAGTACATTG
m*Npas4*	AGGGTTTGCTGATGAGTTGC
m*Usf1*	CCCCCTCACAGAGAGATGAA
m*NR1*	GCTTTTGCAGCCGTGAAC
m*NR2A*	ATTCAACCAGAGGGGCGTA
m*NR2B*	CTTCCTCCTTGCTTTCCACTT
m*NR2C*	AGTTCGGGGGACAGACAAG
m*NR2D*	TGCGATACAACCAGCCAAG
m*ActB*	AAGGCCAACCGTGAAAAGAT

Supplementary Table SI. Primers used for quantitative PCR analysis.
