# Supplementary material for: The CB1 cannabinoid receptor signals striatal neuroprotection via a PI3K/Akt/mTORC1/BDNF pathway
Source: Cell Death Differ. 2015 Feb 20;22(10):1618–29. doi: 10.1038/cdd.2015.11 (PMC4563779; doi:10.1038/cdd.2015.11)
Supplement: Supplementary Figure S1 [file cdd201511x2.pdf]

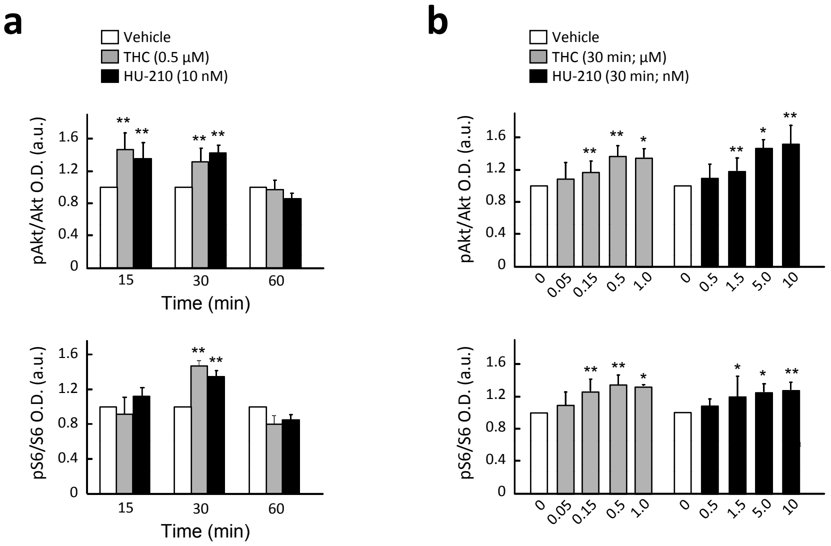

Supplementary Figure S1. Cannabinoids induce Akt and ribosomal S6 protein phosphorylation in a time- and dose-dependent manner. STHdh$^{Q7/Q7}$ cells were incubated (**a**) for the times indicated with vehicle, 0.5 µM THC or 10 nM HU-210, or (**b**) for 30 min with vehicle or the doses indicated of THC or HU-210. Cells were lysed and Western blot analyses were conducted. Quantification of mean optical density (O.D.) values relative to those of α-tubulin are shown (n=3-4 experiments). Data were analyzed using ANOVA with *post hoc* Student-Neuman-Keuls test. *P<0.05, **P<0.01 from the respective vehicle-treated cells.
