# Supplementary material for: The CB1 cannabinoid receptor signals striatal neuroprotection via a PI3K/Akt/mTORC1/BDNF pathway
Source: Cell Death Differ. 2015 Feb 20;22(10):1618–29. doi: 10.1038/cdd.2015.11 (PMC4563779; doi:10.1038/cdd.2015.11)
Supplement: Supplementary Figure S2 [file cdd201511x3.pdf]

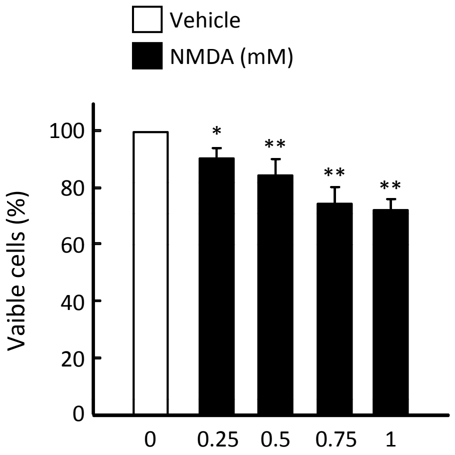

Supplementary Figure S2. NMDA induces the death of STHdh$^{Q7/Q7}$ cells in a dose-dependent manner. STHdh$^{Q7/Q7}$ cells were preincubated for 5 h in Locke's solution with or without the indicated doses of NMDA, and subsequently incubated for 24 h in NMDA-free medium. Relative cell viability is shown (n=4 experiments). Data were analyzed using ANOVA with *post hoc* Student-Neuman-Keuls test. *P<0.05, **P<0.01 from vehicle-treated cells.
