# Supplementary material for: The CB1 cannabinoid receptor signals striatal neuroprotection via a PI3K/Akt/mTORC1/BDNF pathway
Source: Cell Death Differ. 2015 Feb 20;22(10):1618–29. doi: 10.1038/cdd.2015.11 (PMC4563779; doi:10.1038/cdd.2015.11)
Supplement: Supplementary Figure S3 [file cdd201511x4.pdf]

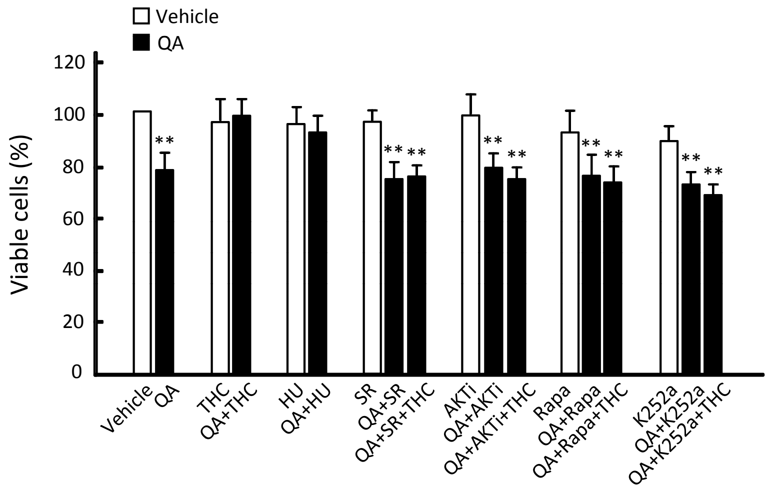

Supplementary Figure S3. The CB_1 cannabinoid receptor protects cultured striatal cells from quinolinic acid-induced excitoxicity via PI3K/Akt/mTORC1/BDNF. STHdh$^{Q7/Q7}$ cells were incubated for 24 h with or without 2.5 mM quinolinic acid (QA) together with vehicle, 0.5 µM THC, 10 nM HU-210, 0.25 µM SR141716, 0.1 µM Akti-1/2, 30 nM rapamicin and/or 25 nM K252a. Relative cell viability is shown (n=4-6 experiments). Data were analyzed using ANOVA with *post hoc* Student-Neuman-Keuls test. ***P*<0.01 from vehicle-treated cells.
