# Supplementary material for: The CB1 cannabinoid receptor signals striatal neuroprotection via a PI3K/Akt/mTORC1/BDNF pathway
Source: Cell Death Differ. 2015 Feb 20;22(10):1618–29. doi: 10.1038/cdd.2015.11 (PMC4563779; doi:10.1038/cdd.2015.11)
Supplement: Supplementary Figure S4 [file cdd201511x5.pdf]

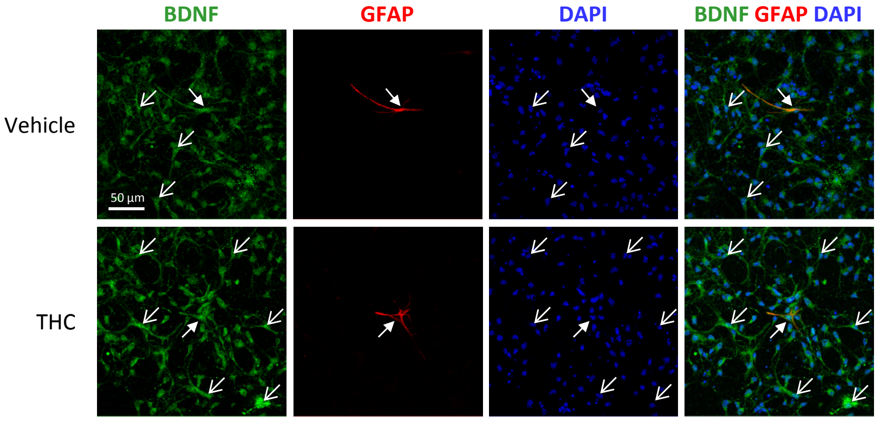

Supplementary Figure S4. Primary cultures of mouse striatal neurons contain very few BDNF/GFAP-double-positive cells. Cultures of primary mouse striatal neurons were incubated for 24 h with vehicle or 0.3 μM THC. Samples were fixed, and BDNF and GFAP immunoreactivities were analyzed. Representative images are shown (n=3 cultures). Close arrows point to $BDNF^+/GFAP^+$ cells, and open arrows point to a selection of $BDNF^+/GFAP^-$ cells. Note the marginal occurrence of $BDNF^+/GFAP^+$ cells.
