# Supplementary material for: The CB1 cannabinoid receptor signals striatal neuroprotection via a PI3K/Akt/mTORC1/BDNF pathway
Source: Cell Death Differ. 2015 Feb 20;22(10):1618–29. doi: 10.1038/cdd.2015.11 (PMC4563779; doi:10.1038/cdd.2015.11)
Supplement: Supplementary Figure S5 [file cdd201511x6.pdf]

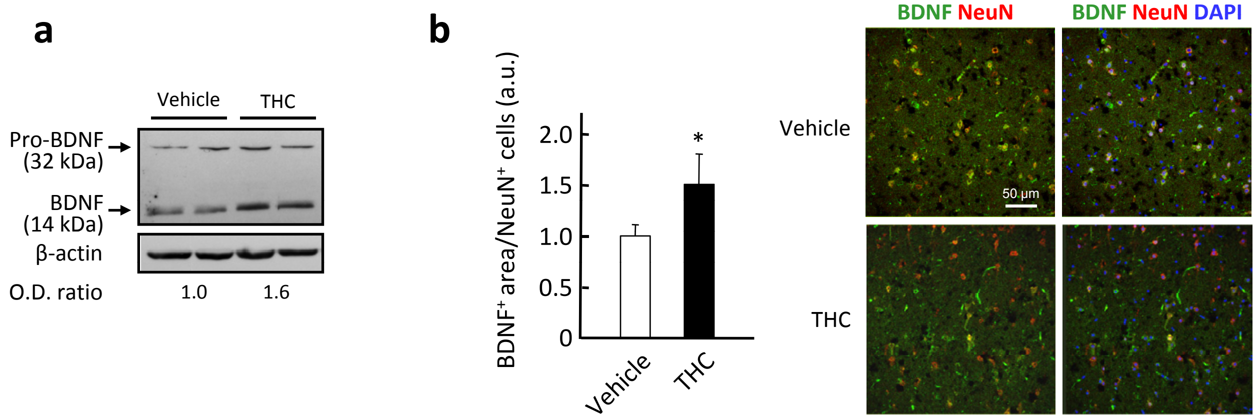

Supplementary Figure S5. THC induces BDNF expression in mouse corticostriatal slices. Mouse corticostriatal organotypic cultures were incubated for 24 h with vehicle or 0.5 µM THC, and BDNF expression was determined in the striatum by Western blot and immunofluorescence analyses. (**a**) A representative blot is shown [n=3 experiments; mean optical density (O.D.) values relative to those of β-actin are given]. (**b**) A representative immunostaining is shown, together with the quantification of the relative values of BDNF-positive area/NeuN-positive cells (n=3 experiments). Data were analyzed using unpaired Student's *t* test. *P<0.05 from vehicle-treated slices.
