# Supplementary material for: The CB1 cannabinoid receptor signals striatal neuroprotection via a PI3K/Akt/mTORC1/BDNF pathway
Source: Cell Death Differ. 2015 Feb 20;22(10):1618–29. doi: 10.1038/cdd.2015.11 (PMC4563779; doi:10.1038/cdd.2015.11)
Supplement: Supplementary Figure S6 [file cdd201511x7.pdf]

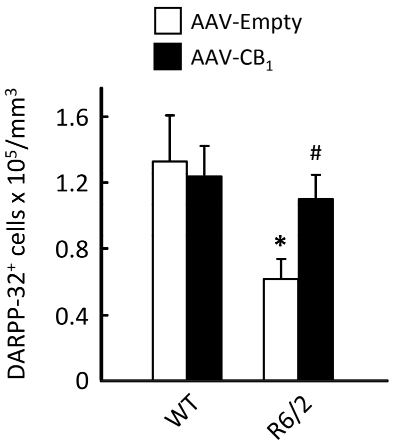


**Supplementary Figure S6. Enforced re-expression of the $CB_1$ receptor normalizes DARPP-32 expression in the striata of R6/2 mice.** R6/2 mice (3.5-4 week-old) and WT littermates were injected stereotactically into the dorsolateral striatum with a recombinant adeno-associated virus (AAV) encoding HA-tagged $CB_1$ receptor or the empty vector as control (n=4-5 animals per group). At week 8 of age animals were sacrificed for stereological counting of DARPP-32-positive cells in the infected region. Data were analyzed using unpaired Student's *t* test. $^{*}P<0.05$ from the WT-Empty group; $^{\#}P<0.05$ from the R6/2-Empty group.
