# Supplementary material for: The CB1 cannabinoid receptor signals striatal neuroprotection via a PI3K/Akt/mTORC1/BDNF pathway
Source: Cell Death Differ. 2015 Feb 20;22(10):1618–29. doi: 10.1038/cdd.2015.11 (PMC4563779; doi:10.1038/cdd.2015.11)
Supplement: Supplementary Figure S7 [file cdd201511x8.pdf]

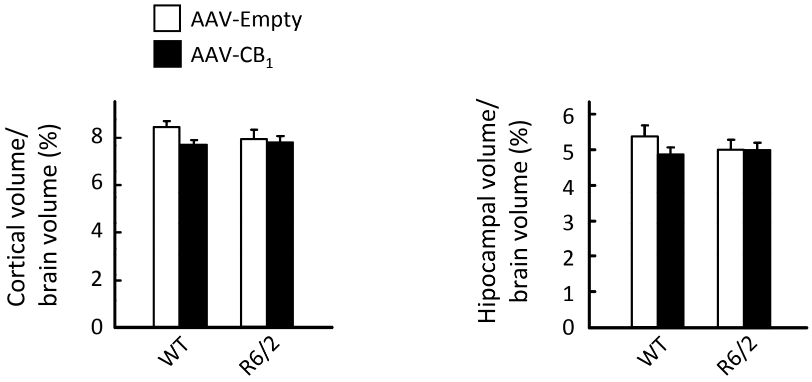

Supplementary Figure S7. Cortical and hippocampal volume does not change in 8 week-old R6/2 mice. R6/2 mice (3.5-4 week-old) and WT littermates were injected stereotactically into the dorsolateral striatum with a recombinant adeno-associated virus (AAV) encoding HA-tagged CB_1 receptor or empty vector as control (n=10-12 animals per group). The volume of the cortex (somatosensory and motor areas) and the hippocampus relative to total brain volume of 8 week-old animals is represented. Data were analyzed using unpaired Student's *t* test.
