# Supplementary material for: The CB1 cannabinoid receptor signals striatal neuroprotection via a PI3K/Akt/mTORC1/BDNF pathway
Source: Cell Death Differ. 2015 Feb 20;22(10):1618–29. doi: 10.1038/cdd.2015.11 (PMC4563779; doi:10.1038/cdd.2015.11)
Supplement: Supplementary Figure S8 [file cdd201511x9.pdf]

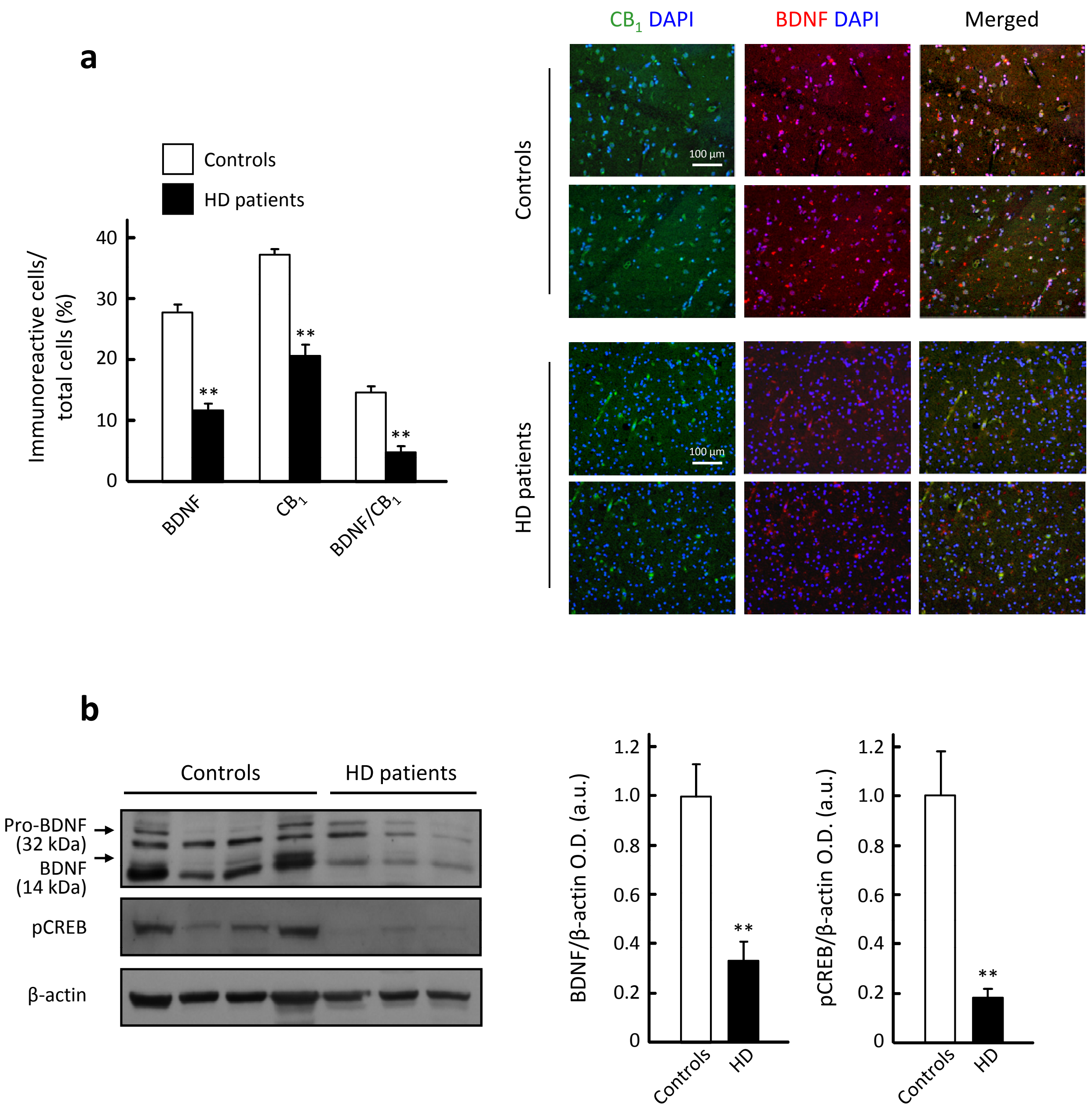

Supplementary Figure S8. The CB_1 receptor and BDNF are down-regulated in *post-mortem* caudate-putamen specimens of HD patients. (**a**) Quantification by immunofluorescence analysis of CB_1/BDNF expression in control subjects (n=7) and HD patients (grades 3-4; n=9). Representative images of CB_1 receptor and BDNF immunostaining in 2 control subjects and 2 HD patients are shown. (**b**) Western blot analysis of BDNF and pCREB expression in control subjects (n=5) and HD patients (grades 3-4; n=4). Quantification of mean ± SEM optical density (O.D.) values relative to those of β-actin as well as representative blots of 4 control subjects and 3 HD patients are shown. Data were analyzed using unpaired Student's *t* test. $^{**}P<0.01$ from the corresponding control group.
